# Case Report: Electrical impedance tomography-guided ultra-protective lung ventilation during veno-venous extracorporeal membrane oxygenation support for fat embolism syndrome complicated by severe acute respiratory distress syndrome

**DOI:** 10.3389/fmed.2026.1891427

**Published:** 2026-09-23

**Authors:** Jiahui Meng, Meng Wu, Jianhua Hu, Yaxin Ning, Danqiong Wang

**Affiliations:** 1 Department of Critical Care Medicine, The Quzhou Affiliated Hospital of Wenzhou Medical University, Quzhou People's Hospital, Quzhou, Zhejiang, China; 2 Quzhou Key Laboratory of Critical Respiratory Mechanics, Quzhou, China

**Keywords:** acute respiratory distress syndrome, case report, electrical impedance tomography, fat embolism syndrome, respiratory mechanics, ultra-protective lung ventilation strategy, venovenous extracorporeal membrane oxygenation

## Abstract

**Background:**

Fat embolism syndrome (FES) is a rare complication of severe multiple trauma. Electrical impedance tomography (EIT) is a noninvasive, real-time, bedside tool for monitoring regional pulmonary ventilation. Veno-venous extracorporeal membrane oxygenation (VV-ECMO) may improve outcomes in critically ill patients in whom conventional mechanical ventilation fails. Ultra-protective lung ventilation strategy (ULPVS) uses very low tidal volumes to markedly reduce lung stress and mitigate ventilator-induced lung injury (VILI).

**Case presentation:**

In June 2025, a 57-year-old woman was admitted to the Department of Orthopedics with a femoral shaft fracture following a motor vehicle accident. Severe respiratory distress developed postoperatively, and she was urgently transferred to the ICU. Based on laboratory findings, chest CT findings, and Oil Red O staining of bronchoalveolar lavage fluid (BALF), FES complicated by severe acute respiratory distress syndrome (ARDS) was considered. In addition to conventional treatment, VV-ECMO was initiated to provide gas-exchange support. An ULPVS was implemented during the early phase of ventilation to reduce the risk of VILI, and EIT was used to guide ventilatory management and titration of the optimal Positive end-expiratory pressure (PEEP) Following these interventions, static lung compliance increased from 46 mL/cmH_2_O to 67.1 mL/cmH_2_O. VV-ECMO support lasted 62 h, and the total duration of mechanical ventilation was 113 h. After stabilization, the patient was transferred back to the Department of Orthopedics and was ultimately discharged in good condition.

**Conclusion:**

Bedside EIT enables dynamic assessment of pulmonary status and may assist in optimizing ventilator settings, providing a useful reference for the management of FES complicated by severe ARDS.

## 1 Introduction

The pathogenesis of FES remains unclear, but two main theories are widely accepted (1), and the diagnosis of FES is primarily based on the Gurd and Wilson criteria (2). Here, we report a woman who developed respiratory distress after surgery for a long-bone fracture and was considered to have FES complicated by ARDS. The clinical course and treatment are described below, and the key diagnostic and therapeutic time points are summarized in Table 1.

**Table 1:** Key clinical events from trauma admission and surgery to fat embolism syndrome, ICU respiratory/organ support, discharge, and follow-up.

Day	Event	Details
Admission day	Admitted to orthopedics after trauma	Left lower-limb pain and swelling with limited mobility; x-ray showed a left femoral shaft fracture
Hospital day 3 (ICU admission)	12:06–14:05 Fracture surgery	Uneventful surgery
	14:25 Cardiorespiratory deterioration	Intubated and transferred to the ICU
	Investigations	Bedside chest x-ray: typical “snowstorm” sign; Hb 103 g/L, PLT 165 × 10^9^/L; Gr 51.5 μmol/L PaO_2_/FiO_2_ 51 mmHg
	Bedside EIT monitoring	Optimal Peep titration; pulmonary perfusion imaging
	Prone positioning for 1 h	Stopped early due to hemodynamic instability
	Persistent refractory hypoxemia and severe lung injury	18:30 VV-ECMO initiated
	Severe metabolic acidosis	CRRT initiated
	22:33 Bronchoscopy	Oil Red O staining of BALF detected fat particles
	Ultra-protective lung ventilation initiated	Tidal volume 230 mL, PEEP 15 cmH_2_O
ICU day 3	Bedside EIT monitoring	Optimal PEEP titration
	Physical examination and laboratory findings	No skin ecchymosis; maximum temperature 38.0 ℃, Hb 67 g/L, PLT 91 × 10^9^/L, Gr 44 μmol/L
ICU day 4	VV-ECMO weaning trial	Successfully weaned
	Follow-up chest x-ray	Bilateral pulmonary infiltrates largely resolved
	Physical examination and laboratory findings	No skin ecchymosis; maximum temperature 37.9 ℃, Hb 80 g/L, PLT 48 × 10⁹/L, Gr 39.3 μmol/L
ICU day 5	Adequate urine output on clinical assessment	CRRT suspended
	Physical examination and laboratory findings	No skin ecchymosis; maximum temperature 38.3 ℃, Hb 83 g/L, PLT 47 × 10⁹/L, Gr 41 μmol/L
ICU day 6	Passed SBT and cuff-leak test	Extubated
ICU day 8	Clinically stable	Returned to the orthopedic ward
Hospital day 16	Discharged	Hb 92 g/L, PLT 344 × 10⁹/L, Gr 36 μmol/L
Day 14 after discharge	Follow-up visit	Femoral x-rays showed good fracture healing; chest x-ray showed no obvious abnormality.

## 2 Case report

A 57-year-old woman with no history of underlying disease or long-term medication use was admitted to the Department of Orthopedics on 2025-06-02 because of pain, swelling, and restricted movement of the left lower extremity following trauma. On admission, her height was 158 cm, actual body weight was 70 kg, body temperature was 37.7 °C, heart rate was 76 beats/min, respiratory rate was 20 breaths/min, blood pressure was 146/86 mmHg, and SpO_2_ was 98%. The left lower extremity was swollen and deformed, with mildly decreased distal sensation; the remainder of the physical examination was unremarkable. Anteroposterior and lateral radiographs of the left thigh are shown in Figure 1E, and non-contrast chest CT showed no obvious abnormalities.

**Figure 1:**
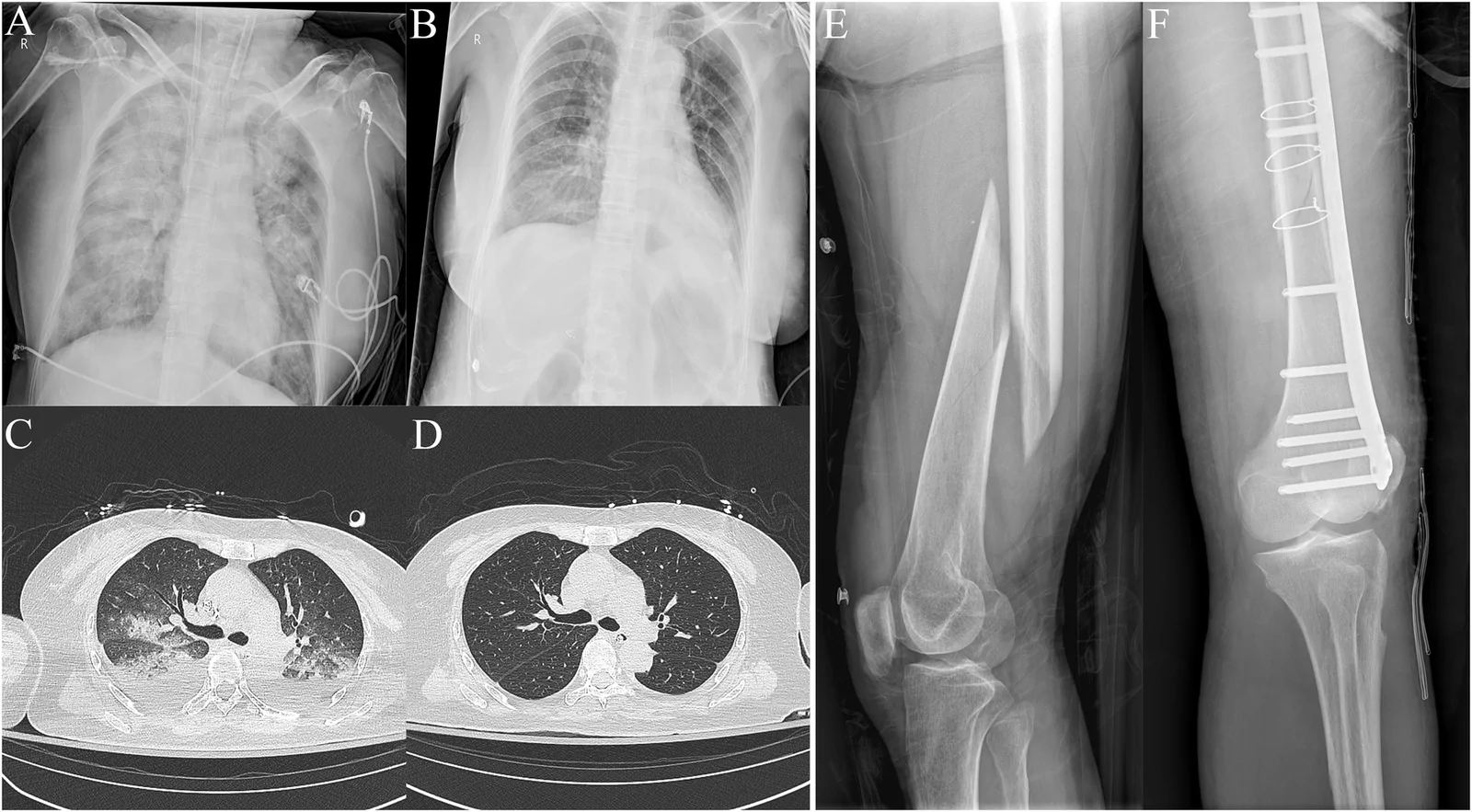
Chest and femoral imaging findings of the patient. **(A,C)** On the day of ICU admission, chest imaging showed the typical “snowstorm” appearance. **(B,D)** On ICU day 4, follow-up chest imaging showed near-complete resolution of bilateral pulmonary infiltrates. **(E)** Radiograph of the left lower extremity on the day of hospital admission showed a left femoral shaft fracture. **(F)** At the 2-week post-discharge follow-up, radiography showed good healing of the femoral fracture.

After contraindications were excluded, surgery was initiated at 12:06 on June 4, 2025. Intraoperative blood loss was approximately 500 mL, blood transfusion volume was approximately 700 mL, and the total fluid infusion volume was 3,100 mL. Norepinephrine was administered intraoperatively to maintain blood pressure, and the operation was completed uneventfully at 14:05. At 14:25, the patient suddenly developed tachypnea; invasive blood pressure decreased to 45/35 mmHg, oxygen saturation decreased to 67%, and heart rate increased to 180 beats/min. Resuscitative measures were initiated immediately, together with endotracheal intubation (7.0-Fr endotracheal tube) and mechanical ventilation. FiO_2_ = 100%, pH = 7.149, PaO_2_ = 54.0 mmHg, PaCO₂ = 56.5 mmHg, and PaO_2_/FiO_2_ = 54 mmHg. The patient had severe respiratory distress and was urgently transferred to the ICU for further resuscitation.

On the night of ICU admission: physical examination showed T 37.9 °C, P 156–168 beats/min, R 41 breaths/min, and BP 109/57 mmHg (maintained with norepinephrine 2.0 ug/min/kg via infusion pump). SpO2 was 82%–90% during invasive mechanical ventilation (A/C mode, PC 15 cmH_2_O, PEEP 15 cmH_2_O, FiO2 100%). Chest CT and chest radiography showed the typical “snowstorm” appearance (Figures 1A,C). EIT-guided titration identified an optimal PEEP of 15 cmH₂O (Figure 2A). Pulmonary perfusion assessment showed ventilation/perfusion matching of 80.37% (Figure 3E), and pulmonary ventilation at that time is shown in Figure 3A. Prone positioning (PP) was initiated at 17:10 but was terminated after 1 h because of hemodynamic instability. Repeat arterial blood gas analysis showed pH = 7.190, PaO_2_ = 51.0 mmHg, PaCO_2_ = 52.4 mmHg, PaO_2_/FiO_2_ = 51 mmHg, and Lac = 3.9 mmol/L. The Murray Lung Injury Score (3) was 3.5, indicating severe lung injury: oxygenation index 51 mmHg (4 points); diffuse pulmonary edema on chest radiography (4 points); PEEP 15 cmH_2_O (3 points); and respiratory system compliance 23.3 mL/cmH₂O (3 points). The patient met the criteria for VV-ECMO initiation. VV-ECMO was initiated at 18:30 that evening (percutaneous cannulation with a 21-Fr cannula for drainage via the right femoral vein and a 17-Fr cannula for return via the right internal jugular vein; rotational speed 2,500 rpm; blood flow 3.8 L/min; air-oxygen blender gas flow 2 L/min). The patient had multiple-organ failure accompanied by severe metabolic acidosis and met the indications for Continuous renal replacement therapy (CRRT). A Baixirui adsorption cartridge was connected to the VV-ECMO circuit; commercially prepared replacement fluid was used at a treatment dose of 25 mL/kg/h. Systemic heparin anticoagulation was administered with a target APTT of 50–70 s, and coagulation parameters were monitored dynamically. Bronchoscopy was performed at 22:33 and revealed abundant yellow frothy secretions. A bronchoalveolar lavage specimen was collected, and BALF Oil Red O staining (4) revealed lipid particles under microscopy (Figure 4). After VV-ECMO flow stabilized, an ULPVS was implemented using volume-controlled ventilation: tidal volume 230 mL and PEEP 15 cmH_2_O. Adequate sedation was provided to suppress spontaneous respiratory effort. An end-inspiratory occlusion maneuver showed a plateau pressure of 20 cmH₂O and a driving pressure of 5 cmH₂O; static lung compliance was 46 mL/cmH₂O. EIT was repeated 2 h after VV-ECMO initiation (Figure 3B).

**Figure 2:**
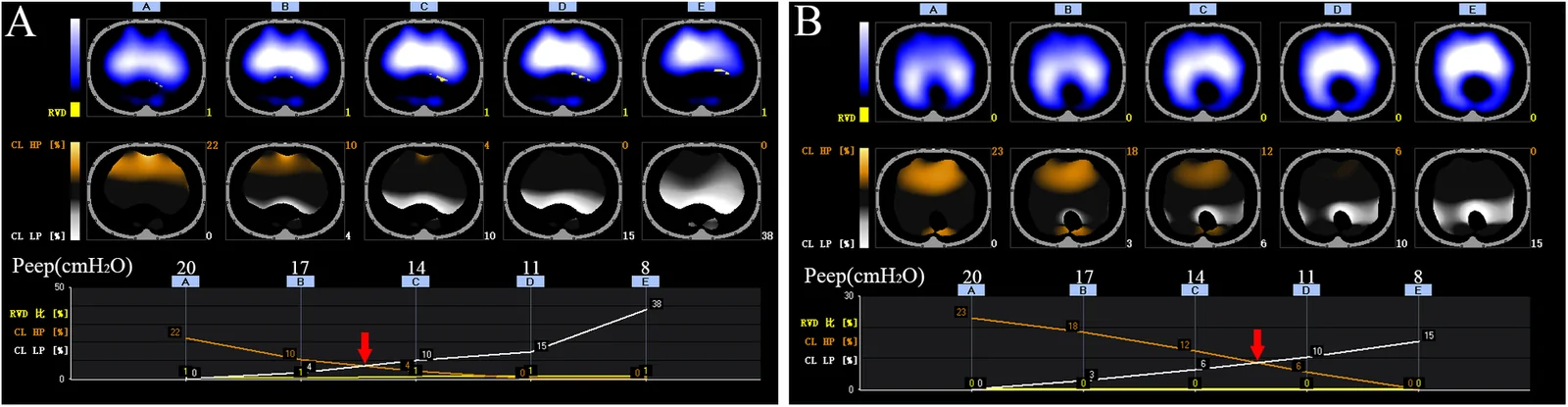
EIT-guided titration of the optimal PEEP. The PEEP value corresponding to the intersection of the orange line (CL HP) and the white line (CL LP) was considered the optimal PEEP. **(A)** On the night of ICU admission, the optimal PEEP was titrated to 15 cmH_2_O. **(B)** On ICU day 3, the optimal PEEP was titrated to 12 cmH_2_O. RVD: Regional Ventilation Delay.

**Figure 3:**
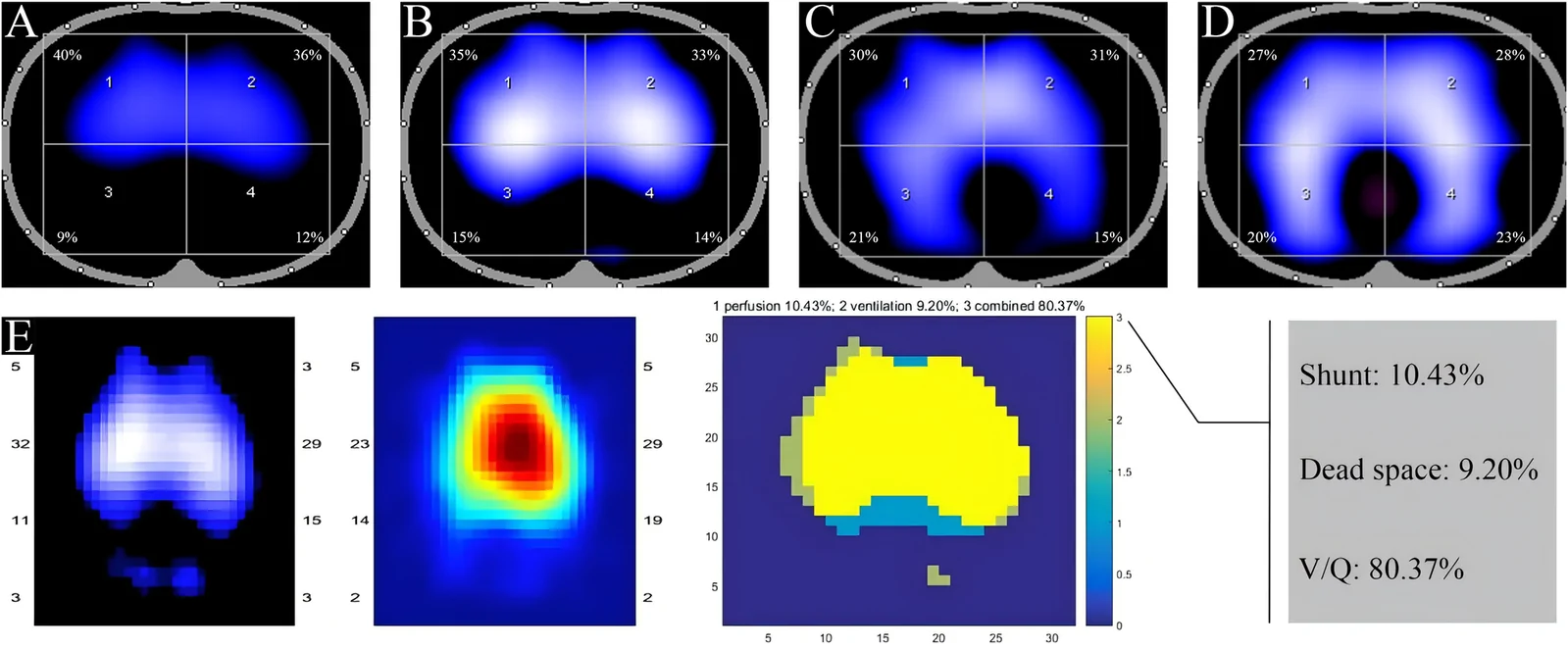
EIT monitoring of pulmonary ventilation and perfusion. **(A)** On the day of ICU admission, before initiation of VV-ECMO. **(B)** Two hours after initiation of VV-ECMO. **(C)** On ICU day 3. **(D)** On the day of ICU discharge. **(E)** EIT-based pulmonary perfusion assessment: left, ventilation map (regions of low ventilation are shown in dark blue, whereas regions of high ventilation are shown from light blue to white); middle, perfusion map (regions of high perfusion are shown in red); right, regional ventilation–perfusion matching map generated by integrating the ventilation and perfusion images. (The color scale of each panel was independently normalized for each measurement; therefore, differences in color intensity should not be directly compared across panels to assess the degree of ventilation. Images are displayed according to the standard radiological orientation: the upper part of each image represents the ventral region and the lower part the dorsal region; the left side of the image corresponds to the patient's right lung and the right side to the patient's left lung. Image orientation remains unchanged regardless of whether the patient is in the supine or prone position.).

**Figure 4:**
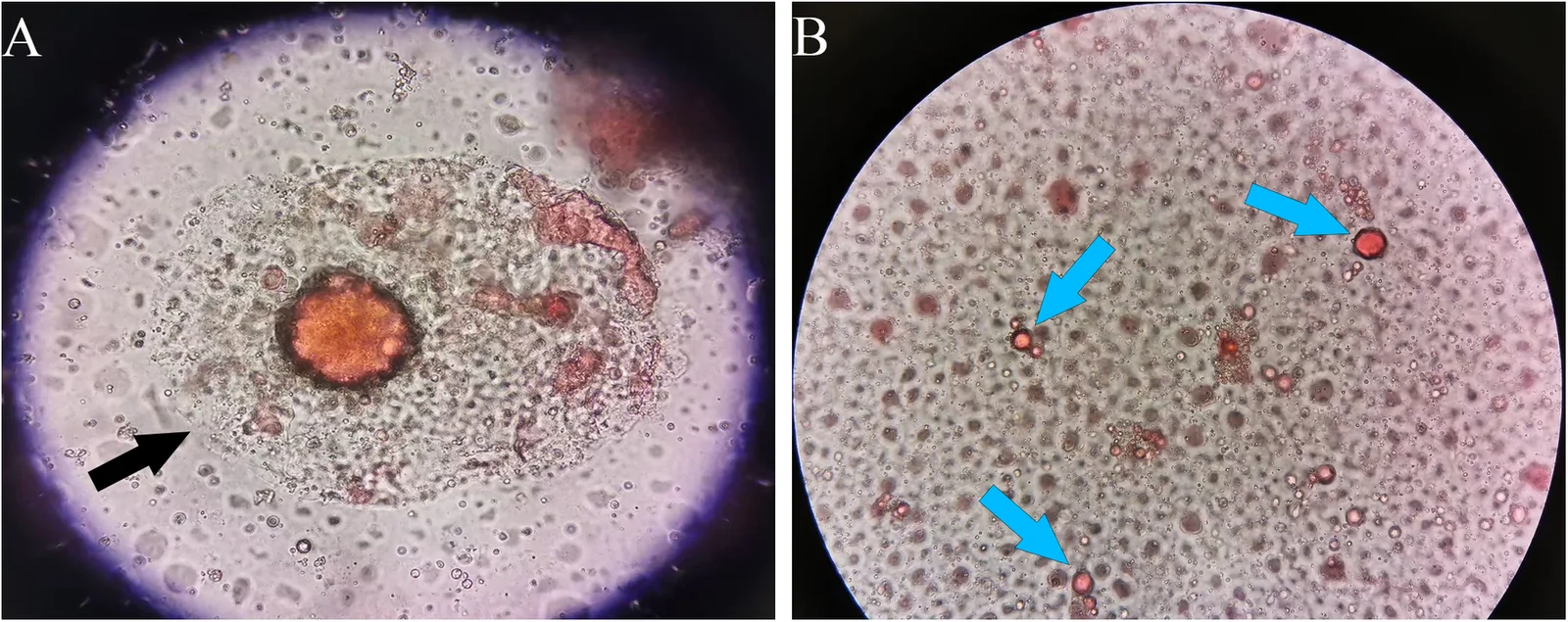
Oil Red O staining of the BALF specimen. **(A)** Macrophages containing orange-stained lipid droplets are clearly visible (black arrows). **(B)** More extracellular lipid droplets are observed (blue arrows).

Day 3: PEEP was retitrated under EIT guidance, and the optimal PEEP was 12 cmH₂O (Figure 2B). Ventilator settings were adjusted to volume-controlled ventilation with a tidal volume of 300 mL and PEEP of 12 cmH₂O. The measured plateau pressure was 17 cmH₂O, the calculated driving pressure was 5 cmH₂O, and static lung compliance was 60 mL/cmH₂O. Pulmonary ventilation assessed by EIT is shown in Figure 3C. The highest body temperature was 38.0 °C, Hb was 67 g/d, and PLT was 91 × 10⁹/L.

Day 4: A VV-ECMO weaning trial was performed. Ventilator settings were adjusted to FiO₂ 30%, PC 9 cmH₂O, and PEEP 10 cmH₂O. VV-ECMO blood flow was maintained at 3.8 L/min, and VV-ECMO sweep gas flow was turned off. Vital signs were monitored during the weaning trial. After 1 h: pH 7.415, PaCO_2_ 36.7 mmHg, PaO_2_ 112 mmHg, SaO_2_ 99.8%, and PaO_2_/FiO_2_ 373 mmHg. After 2 h: pH 7.437, PaCO_2_ 38.1 mmHg, PaO_2_ 106 mmHg, SaO_2_ 99.5%, and PaO_2_/FiO_2_ 353 mmHg. The weaning trial was successful, and VV-ECMO was discontinued. The total duration of VV-ECMO support was 62 h, with no VV-ECMO-related complications. Follow-up chest imaging showed near-complete resolution of bilateral pulmonary infiltrates (Figures 1B,D). Static lung compliance had increased to 67.1 mL/cmH2O. The highest body temperature that day was 37.9 °C, Hb was 80 g/d, and PLT was 48 × 10⁹/L.

Day 5: Following physician assessment, urine output was satisfactory and hemodynamics were stable; therefore, CRRT was discontinued after a total treatment duration of 77 h. The highest body temperature that day was 38.3 °C, Hb was 83 g/d, and PLT was 47 × 10⁹/L.

Day 6: The patient passed the spontaneous breathing trial (SBT) and cuff-leak test. The endotracheal tube was removed, and oxygen was administered via nasal cannula. The total duration of invasive mechanical ventilation was 113 h.

Day 8: The patient's condition was stable, and pulmonary ventilation was reassessed by EIT (Figure 3D). She was transferred to the Department of Orthopedics for continued treatment and subsequently recovered and was discharged.

During the ICU stay, the patient also received meropenem for anti-infective treatment, blood transfusion, acid-suppressive therapy, gastric mucosal protection, and other supportive treatments.

Follow-up: At 1 month after discharge, anteroposterior and lateral radiographs of the femur showed satisfactory fracture healing (Figure 1F), and chest radiography showed no obvious abnormalities.

## 3 EIT monitoring

1. Equipment and measurement (5): A PulmoVista device (Dräger, Germany) was used. An EIT belt equipped with 16 surface electrodes was secured circumferentially around the patient's thorax at the level of the fourth to sixth intercostal spaces and connected to the EIT monitor. Weak high-frequency alternating currents were applied through the surface electrodes surrounding the thorax, and surface voltage signals were acquired. Using a reconstruction algorithm, dynamic changes in electrical impedance of the pulmonary tissue during respiration were converted into images. An increase in gas content results in increased impedance and a brighter image, enabling real-time bedside assessment of regional pulmonary ventilation distribution. EIT data analysis was performed offline using dedicated software (Draeger EIT Data Analysis Tool Ver 6.3; Dräger Medical, Germany).
2. Pulmonary perfusion: At end-expiration, the patient was instructed to hold her breath for ≥8 s, and 10 mL of 10% hypertonic sodium chloride solution was rapidly injected through a central venous catheter. Because of its high conductivity, 10% sodium chloride can serve as a contrast agent for EIT imaging. As it passes through the right heart into the pulmonary circulation, it causes a marked decrease in regional thoracic impedance. Regional time-impedance curves were analyzed using indicator first-pass dilution kinetics (6) to obtain regional pulmonary perfusion information.
3. Optimal PEEP titration: A lung recruitment maneuver was first performed, followed by a decremental PEEP trial starting at 20 cmH₂O, with PEEP reduced by 3 cmH₂O at each step. Each PEEP level was maintained for 5 min until 8 cmH₂O was reached. After titration, the data were imported into the EIT analysis software, and the OverDistension and Collapse (OD-CL) method (7) was applied. The software calculated cumulative compliance loss on the high-pressure side (CL-HP, representing pulmonary overdistension) and cumulative compliance loss on the low-pressure side (CL-LP, representing alveolar collapse). The intersection of the two curves was defined as the optimal PEEP, at which the proportions of overdistension and collapse were considered balanced.

## 4 Discussion

According to the Gurd criteria, this patient met one major criterion (respiratory symptoms and chest radiographic abnormalities) and four minor criteria (arterial PaO₂ < 60 mmHg, tachycardia, thrombocytopenia, and decreased hemoglobin). According to the Schonfeld criteria, the patient scored 10 points: petechial rash (0 points), diffuse pulmonary infiltrates (4 points), hypoxemia (3 points), neurological symptoms (0 points), elevated body temperature (1 point), heart rate > 120 beats/min (1 point), and respiratory rate > 30 breaths/min (1 point); a score > 5 supports the diagnosis. Because the clinical manifestations of FES are nonspecific, the differential diagnoses in this case included aspiration pneumonia, volume overload, pulmonary thromboembolism, pulmonary infection, and transfusion-related acute lung injury.

The patient developed acute respiratory failure within 7 days of exposure to the risk factor. Chest CT showed bilateral diffuse patchy infiltrates that could not be explained by pleural effusion or atelectasis, and echocardiography provided no evidence of cardiogenic pulmonary edema. During invasive mechanical ventilation with PEEP > 5 cmH_2_O, PaO_2_/FiO_2_ remained < 100 mmHg. The patient therefore met the Berlin diagnostic criteria for ARDS (8), and severe ARDS was diagnosed.

VV-ECMO can efficiently remove CO_2_ from the blood and markedly reduce the respiratory metabolic burden, thereby acting synergistically with ULPVS. ULPVS uses very low tidal volumes combined with appropriate PEEP (ultra-low tidal volume 2–4 mL/kg, lower plateau pressure ≤20–25 cmH2O, and appropriate PEEP) to reduce alveolar overdistension and thereby mitigate VILI. Previous studies (9–11) have used heterogeneous tidal-volume settings for “ultra-protective/ultra-low tidal-volume” ventilation, and clinical implementation requires dynamic adjustment according to CO_2_ retention, acid-base status, and respiratory mechanics. In this case, an ULPVS was used initially. To avoid further worsening of hypercapnia with an extremely low tidal volume, the ventilatory parameters were moderately relaxed. The patient's ideal body weight was 50.4 kg, and the tidal volume was set at 230 mL (4.56 mL/kg). As pulmonary function and compliance improved, the tidal volume was moderately increased to promote lung recruitment, and was subsequently adjusted to 300 mL (5.95 mL/kg).

PP (12) can improve the uniformity of pulmonary ventilation and reduce regional heterogeneity of lung injury, thereby improving lung compliance. For patients with ARDS, PP for at least 16 h per day is routinely recommended. In this patient, hemodynamic instability developed after 1 h of PP, and the session had to be terminated. Because the duration of PP was insufficient and EIT monitoring was not performed after termination of the prone session, this case could not assess the effect of PP on pulmonary ventilation.

It should be noted that FES is inherently self-limiting; therefore, the patient's clinical improvement cannot be attributed entirely to interventions such as VV-ECMO and EIT-guided ventilation. These measures primarily provided respiratory support and allowed time for the natural course of the disease, and their potential benefits require validation in additional cases.

## 5 Conclusion

In this patient with post-traumatic FES complicated by severe ARDS, the use of VV-ECMO demonstrates the feasibility of this technique in such critically ill patients. EIT guidance enabled individualized titration of ventilator settings and was associated with short-term improvement in respiratory physiology. Future multicenter observational studies are warranted to evaluate the generalizability of this approach.

## Data Availability

The original contributions presented in the study are included in the article/Supplementary Material, further inquiries can be directed to the corresponding author.
